# Roles of functional catechol-O-methyltransferase genotypes in Chinese patients with Parkinson’s disease

**DOI:** 10.1186/s40035-017-0081-9

**Published:** 2017-04-26

**Authors:** Qin Xiao, Yiwei Qian, Jiujiang Liu, Shaoqing Xu, Xiaodong Yang

**Affiliations:** Ruijin Hospital affiliated to Shanghai JiaoTong University School of Medicine, No. 197, Ruijin Er Road, Shanghai, 200025 China

**Keywords:** Parkinson’s disease, COMT, SNP, Pharmacotherapy

## Abstract

**Background:**

Recent studies have found that the functional catechol-O-methyltransferase (COMT) gene may be associated with the susceptibility to and pharmacotherapy of Parkinson’s disease (PD). In this case–control study, we investigated the most common functional COMT gene haplotypes that had been shown to influence COMT enzymatic activity and the association of the single and combined COMT haplotypes with clinical symptoms and pharmacotherapy in Chinese patients with PD.

**Methods:**

One hundred forty-three patients with idiopathic PD and 157 healthy individuals were enrolled in this study. Four single nucleotide polymorphisms (SNPs) in the COMT gene (formed by SNPs) were genotyped in each participant: rs6269 A > G; rs4633 C > T; rs4818 C > G; and rs4680 G > A.

**Results:**

The frequencies of rs4633 T carriers, rs4680 A carriers and the two linked rs4633-rs4680 T/A carriers were significantly higher in the early onset PD group than in the healthy controls (all *P* < 0.05). Homozygosity for rs4633 (TT), rs4680 (AA) and of the two linked rs4633-rs4680 (TT/AA) was significantly more frequent in patients who exhibited the “wearing-off” phenomenon, longer disease duration, higher levodopa equivalent doses (LED) and higher Unified Parkinson’s Disease Rating Scale (UPDRS) scores (*P* < 0.05). No significant differences were observed in the clinical features of patients who carried individual rs6269 and rs4818, the two linked rs6269-rs4818 and the four combined COMT SNPs.

**Conclusions:**

The results showed a possible association of combined functional COMT SNPs with PD risk, disease duration, the “wearing-off” phenomenon, daily LEDs and higher UPDRS scores, which may be useful in instituting individualized therapy for patients with PD.

**Electronic supplementary material:**

The online version of this article (doi:10.1186/s40035-017-0081-9) contains supplementary material, which is available to authorized users.

## Background

Parkinson’s disease (PD) is a common progressive neurodegenerative disease in middle-aged and elderly people that consists of interactions between environmental and genetic factors and contributes to the dysfunction of dopaminergic neurotransmission in the central nervous system (CNS).

Genetic polymorphisms in enzymes, which regulate the biosynthesis or degradation of dopamine and its metabolites, may play a role in the susceptibility to PD and levodopa treatment. Catechol-O-methyltransferase (COMT) is one of the most important enzymes in the metabolism of drugs and neurotransmitters, such as L-dopa, dopamine, noradrenaline, etc. [1]; COMT-mediated O-methylation inactivates biologically active or toxic catechol and some hydroxylated metabolites [2]. Blocking of COMT activity further reduces peripheral levodopa degradation, as it prolongs plasma half-life of levodopa and elevates delivery of levodopa to the brain [3]. The human COMT gene is located on chromosome 22q11.21, and polymorphisms in COMT are associated with high, intermediate and low levels of enzyme activity, which might affect the risk and treatment of PD [4].

Most studies focused on the rs4680 single nucleotide polymorphisms (SNPs), which is a G to A substitution in the fourth exon of the COMT gene, leading to the substitution of valine 158 with a methionine (Val158Met) and resulting in low COMT enzyme activity, which is regarded as the L (low activity) allele, in contrast to the H (high activity) allele [5]. The COMT protein encoded by the L allele is thermolabile, which influences individual variations in the therapeutic response to levodopa and susceptibility to PD [6–9]. Moreover, studies [1, 10–12] have focused on three other common haplotypes comprising combinations of four SNPs in the COMT gene: rs6269 A > G, rs4633 C > T (His62His), rs4818 C > G (Leu136Leu) and rs4680 G > A (Val158Met). The haplotype structure formed by all four SNPs affects COMT enzymatic activity more than the single rs4680 SNP, which was regarded as a key determinant of COMT enzymatic activity in previous studies. COMT activity has been reported to depend on the presence of haplotypes formed by the four analyzed SNPs: A_C_C_G-low activity-L, A_T_C_A –medium activity-M, and G_C_G_G-high activity-H [11]. One study in Poland reported a high frequency of patients with late onset PD who were carriers of the G_C_G_G (high activity) haplotype, and the COMT haplotype seemed to have little influence on the development of levodopa-induced dyskinesia [1].

Additionally, some of the SNPs that have been studied with respect to PD risk exhibit linkage disequilibrium, although interethnic differences exist. According to the 1000 genome datasets (http://browser.1000genomes.org/Homo_sapiens/Search/Results?site=ensembl&q=comt), Caucasian individuals show higher linkage values: the SNPs rs4680 and rs4633 exhibit complete linkage (D’ = 1.00), the SNPs rs4680 and rs4818 exhibit partial linkage (D’ = 0.685), and the SNPs rs4633 and rs4818 also exhibit partial linkage (D’ = 0.685). However, for Oriental individuals, all linkages showed lower values: D’ = 0.927 for the SNPs rs4680 and rs4633, D’ = 0.240 for the SNPs rs4680 and rs4818, and D’ = 0.223 for the SNPs rs4633 and rs4818 [4]. To our knowledge, no study has examined the associations between the two linked COMT SNPs (rs4680-rs4633 or rs6269-rs4818) with PD risk, clinical characteristics, and drug treatment. However, of the studies that have estimated the association between the combined two linked or four combined COMT SNPs and PD risk, the clinical symptoms and pharmacotherapy have not yet been investigated in China.

In this case–control study, we investigated the association of the most common functional COMT gene haplotypes (formed by SNPs: rs6269 A > G; rs4633C > T; rs4818 C > G; and rs4680 G > A), whether single, two linked or four combined SNPs, with PD risk in China. In addition, we examined the association of single, two linked or four combined COMT SNPs with the patients’ clinical features and pharmacotherapy, particularly the complications of levodopa therapy in patients with PD.

### Methods

#### Samples

We recruited 143 outpatients with idiopathic PD (79 males, 64 females, mean age (standard deviation [SD]) 65.1 (8.7)) as the PD group from the Movement Disorder Clinic at the Department of Neurology of Ruijin Hospital (affiliated with the Shanghai JiaoTong University School of Medicine, Shanghai, China) and 157 age- and gender-matched healthy individuals (88 males, 69 females, mean age (SD) 65.4 (7.2)) as the control group during the period from December 2013 to December 2014. All 143 patients with PD who were eligible for this study were diagnosed with idiopathic PD according to the UK brain bank criteria [13]. Among the 143 patients with PD, 24 were diagnosed with early onset PD (EOPD, onset before 50 years of age) and 119 were diagnosed with late onset PD (LOPD, onset after 50 years) [1]. All individuals who were diagnosed with atypical parkinsonism, including progressive supranuclear palsy (PSP), multiple system atrophy (MSA), vascular or drug-induced parkinsonism and severe dementia, characterized by a Mini Mental State Examination (MMSE) score <24 [14], were excluded from our study.

Each participant was informed of the purpose of this study and signed an informed consent form. This study was performed in accordance with The Code of Ethics of the World Medical Association (Declaration of Helsinki) for experiments involving humans and the protocol was approved by the Research Ethics Committee of Ruijin Hospital affiliated with the Shanghai Jiao Tong University School of Medicine.

#### Genotyping

Genomic DNA was extracted from peripheral blood samples collected from each individual using the standardized phenol/chlorine extraction method. Genotyping of rs6269 A > G was performed on a 456-bp DNA fragment amplified with the following primers: forward: CAACAGCCTGAGTCCGTGTC, reverse: TCCAGCCGATAAGGCACAGG. rs4680 G > A and rs4818 C > G were contained within the same 564-bp DNA fragment and amplified with the following primers: forward: ACCAGCGTGAGCATAGAGGC, reverse: GGTTTTCAGTGAACGTGGTGTG. rs4633C > T was genotyped using a 398-bp DNA fragment amplified with following pair of primers: forward: CTTGCCCCTCTGCAAACAC, reverse: TTCTTGTCGCCCACGTTC. A 50 μl volume was used to conduct the polymerase chain reaction (PCR), which contained 1 μl of 100 ng/μl primer, 0.6 μl 5 of U/ml r-Taq DNA polymerase, 1 μl of 10 mM dNTPs, 5 μl of 10 × Buffer, 2.5 μl of genomic DNA and 38.9 μl of ddH2O. For rs6269, the PCR thermal profile consisted of initial denaturation at 95 °C for 5 min, 30 cycles at 95 °C, 58 °C and 72 °C (each step for 30 s) and a final elongation at 72 °C for 7 min. For rs4680 and rs4818, the PCR conditions included an initial step at 95 °C for 5 min, 30 cycles at 95 °C for 30 s, 58 °C for 30 s and 72 °C for 40 s and a final elongation at 72 °C for 7 min. For rs4633, the PCR conditions included an initial step at 95 °C for 5 min, 30 cycles at 95 °C, 56 °C and 72 °C (each step for 30 s) and a final elongation at 72 °C for 7 min. The PCR products were purified and sequenced on an ABI 3730xl automated sequencer (Applied Biosystems, Foster City, CA, USA).

#### Data collection

In this study, clinical data were collected through face-to-face interviews and questionnaires assessing demographic and clinical characteristics of patients with PD that were conducted by at least two movement disorder specialists. Demographic information included age, gender, and employment information; PD clinical characteristics included disease duration, onset condition (such as the age at diagnosis, the initial limb in which the PD symptoms were observed, and the first symptom of PD), drug treatment, and motor complications (mainly include “wearing-off” phenomenon, characterized by gradual expected re-emergence of parkinsonian symptoms at the end of an L-dopa dose, and L-dopa–induced dyskinesia, characterized by emergence of hyperkinetic involuntary movements). All patients were examined during the “on” state (improvement in symptoms after L-dopa administration is described as being “on”) to determine their baseline motor function and activities of daily living using the Unified Parkinson’s Disease Rating Scale (UPDRS), and disease severity was evaluated using the classification of Hoehn and Yahr stage (H&Y stage). The levodopa equivalent doses (LED) were calculated using a method reported in a previous study [15]. The PD-related non-motor symptoms were evaluated using the Non-Motor Symptoms questionnaire for Parkinson's disease (NMS-Quest), the Hamilton Anxiety Scale (HAMA), and the Hamilton Depression Scale (HAMD).

#### Statistical analysis

In this study, SPSS 17.0 software (SPSS Inc., Chicago, IL, USA) was used for the statistical analysis. In descriptive analyses, mean and standard deviation (mean ± SD) was used for normally distributed continuous variables, median and interquartile range (median ± IQ) for continuous variables with skewed distribution, and proportions for categorical variables. Concordance of genotype distribution with Hardy–Weinberg equilibrium was assessed using the Chi-square test. The Chi-square test and Fisher’s exact test were used to compare the COMT allelic frequencies or genotype frequencies between the control group and PD group. Analysis of variance of factorial design was used perform the association between COMT polymorphisms and clinical outcomes, adjusted age and sex as covariates. Binary logistic regression model was used to analyze the relation between SNP and motor complication (wearing-off phenomenon and dyskinesia), with age and sex as covariates. *P*-values were adjusted with Bonferroni correction. All statistically significant differences were considered *P*-value < 0.05.

## Results

### Linkage disequilibrium and haplotype analysis

In the pairwise analysis of the four COMT SNPs using the Haploview software, rs6269, rs4633, rs4818 and rs4680 showed strong linkage disequilibrium (LD) in both groups (Table 1), particularly rs6269-rs4818 and rs4633-rs4680. The LD of rs6269-rs4818 in the control group was D’ = 0.94, LOD = 48.14, *r*2 = 0.85, whereas the LD in the PD group was D’ = 0.98, LOD = 48.84, *r*2 = 0.92. The LD of rs4633-rs4680 in the control group was D’ = 0.95, LOD = 41.80, *r*2 = 0.83, whereas the LD in the PD group was D’ = 1.00, LOD = 49.53, *r*2 = 0.96. The patients with PD showed a stronger linkage in rs6269-rs4818 and rs4633-rs4680; therefore, we used the two linked SNPs rs6269-rs4818 and rs4633-rs4680 in the subsequent analysis.

**Table 1:** Linage disequilibrium (LD) of the combined studied SNPs in PD patients and healthy controls

		Control group			PD group		
		rs4633	rs4818	rs4680	rs4633	rs4818	rs4680
rs6269	*D’*	0.90	0.94	0.77	0.91	0.98	1.00
	*LOD*	8.05	48.14	4.84	4.43	48.84	5.49
	*r* ^*2*^	0.18	0.85	0.12	0.14	0.92	0.16
rs4633	*D’*	-	1.00	0.95	-	0.91	1.00
	*LOD*	-	10.20	41.80	-	3.85	49.53
	*r* ^*2*^	-	0.21	0.83	-	0.13	0.96
rs4818	*D’*	-	-	0.94	-	-	1.00
	*LOD*	-	-	7.47	-	-	4.88
	*r* ^*2*^	-	-	0.17	-	-	0.16

The LD pattern of the studied COMT SNPs was analyzed in Haploview. D' and r^2^: measures of linkage disequilibrium between two genetic markers. *LOD* the logarithm of the odds for LD

### Different COMT SNPs influenced the susceptibility to PD

The genotype and allele distributions of COMT SNPs were listed on Table 2. All the observed genotype or allele frequencies did not differ from the expected frequencies according to the Hardy-Weinberg equilibrium. For the single SNPs (rs6269A > G; rs4633C > T; rs4818C > G and rs4680G > A), no significant differences were found in the allele and genotype frequencies between the PD group and the control group. However, the frequency of rs6269 GG (*P* = 0.09) and allele G (*P* = 0.09), rs4818 CG + GG (*P* = 0.09) and allele G (*P* = 0.08) tended to be slightly lower among patients with PD than the controls. For the two linked SNPs (rs4633-rs4680 and rs6269-rs4818), there were also no significant differences in the allele and genotype frequencies between the PD group and control group. Moreover, no significant differences in the four combined SNPs (rs6269, rs4633, rs4818, and rs4680) were observed between the two groups. However, the frequency of the low (A_C_C_G) activity haplotypes (L) tended to be slightly higher among patients with PD (*P* = 0.10, G_C_G_G-high activity haplotype (H) as reference), and the frequency of the haplotypes without the H carrier tended to be higher in the PD group than in the controls (*P* = 0.10, H carrier was used as a reference).

**Table 2:** Frequencies of the studied SNPs in PD, EOPD and LOPD patients and healthy controls

	Control group n (%)	PD group *n* (%)	*P*	OR (95% CI)	EOPD *n* (%)	*P*	OR (95% CI)	LOPD *n* (%)	*P*	OR (95% CI)
rs6269 A > G										
AA	56 (35.0)	62 (43.4)	-	-	10 (41.7)	-	-	52 (43.7)	-	-
AG	77 (49.0)	67 (46.9)	0.33	0.79 (0.48–1.28)	12 (50.0)	0.77	0.87 (0.35–2.16)	55 (46.2)	0.32	0.77 (0.46–1.28)
GG	24 (15.9)	14 (9.9)	0.09	0.53 (0.25–1.12)	2 (8.3)	0.50	0.47 (0.10–2.29)	12 (10.1)	0.12	0.54 (0.25–1.19)
AG + GG	101 (65.0)	81 (56.6)	0.17	0.72 (0.46–1.15)	14 (58.3)	0.57	0.78 (0.32–1.86)	67 (56.3)	0.18	0.71 (0.44–1.16)
Major (A) allele frequency	189 (60.2)	191 (66.8)	-	-	32 (66.7)	-	-	159 (66.8)	-	-
Minor (G) allele frequency	125 (39.8)	95 (33.2)	0.09	0.75 (0.54–1.05)	16 (33.3)	0.29	0.71 (0.38–1.34)	79 (33.2)	0.11	0.75 (0.53–1.07)
rs4633 C > T										
CC	89 (55.4)	78 (54.6)	-		8 (33.3)	-	-	70 (58.8)	-	-
CT	57 (37.2)	56 (39.2)	0.64	1.12 (0.70–1.81)	15 (62.5)	0.02*	2.93 (1.17–7.35)	41 (34.5)	0.73	0.92 (0.55–1.52)
TT	11 (7.4)	9 (6.3)	0.89	0.94 (0.37–2.37)	1 (4.2)	1.00	1.01 (0.12–8.87)	8 (6.7)	0.87	0.93 (0.35–2.42)
CT + TT	68 (44.6)	65 (45.5)	0.71	1.09 (0.69–1.72)	16 (66.7)	0.03*	2.68 (1.06–6.47)	49 (41.2)	0.72	0.92 (0.57–1.49)
Major (C) allele frequency	235 (74.8)	212 (74.1)	-	-	31 (64.6)	-	-	181 (76.1)	-	-
Minor (T) allele frequency	79 (25.2)	74 (25.9)	0.84	1.04 (0.72–1.50)	17 (35.4)	0.13	1.63 (0.86–3.11)	57 (23.9)	0.75	0.94 (0.63–1.39)
rs4818 C > G										
CC	55 (35.0)	64 (44.8)	-	-	10 (41.7)	-	-	54 (45.4)	-	-
CG	82 (52.2)	66 (46.2)	0.14	0.69 (0.43–1.12)	12 (50.0)	0.64	0.81 (0.33–1.99)	54 (45.4)	0.12	0.67 (0.40–1.12)
GG	20 (12.7)	13 (9.1)	0.14	0.56 (0.26–1.23)	2 (8.3)	0.72	0.55 (0.11–2.73)	11 (9.2)	0.17	0.56 (0.25–1.28)
CG + GG	102 (65.0)	79 (55.3)	0.09	0.67 (0.42–1.06)	14 (58.3)	0.53	0.76 (0.32–1.81)	65 (54.6)	0.08	0.65 (0.40–1.06)
Major (C) allele frequency	192 (61.2)	194 (67.8)	-	-	32 (66.7)	-	-	162 (68.1)	-	-
Minor (G) allele frequency	122 (38.9)	92 (32.2)	0.09	0.75 (0.53–1.05)	16 (33.3)	0.46	0.79 (0.41–1.50)	76 (31.9)	0.09	0.74 (0.52–1.05)
rs4680 G > A										
GG	91 (58.0)	79 (55.2)	-	-	8 (33.3)	-	-	71 (59.7)	-	-
AG	57 (36.3)	56 (39.2)	0.61	1.13 (0.70–1.82)	15 (62.5)	0.02*	2.99 (1.19–7.51)	41 (34.5)	0.75	0.92 (0.56–1.53)
AA	9 (5.7)	8 (5.6)	0.96	1.02 (0.38–2.78)	1 (4.2)	0.59	1.26 (0.14–11.28)	7 (5.9)	1.00	1.00 (0.35–2.81)
AG + AA	66 (42.0)	64 (44.8)	0.64	1.12 (0.71–1.77)	16 (66.7)	0.02*	2.76 (1.12–6.82)	48 (40.3)	0.78	0.93 (0.57–1.51)
Major (G) allele frequency	239 (76.1)	214 (74.8)	-	-	31 (64.6)	-	-	183 (76.9)	-	-
Minor (A) allele frequency	75 (23. 9)	72 (25.2)	0.71	1.07 (0.74–1.56)	17 (35.4)	0.09	1.75 (0.92–3.33)	55 (23.1)	0.83	0.96 (0.64–1.43)
rs4633 C > T; rs4680 G > A										
CC/GG	86 (54.8)	78 (54.5)	*-*	-	8 (33.3)	-	-	70 (58.8)	-	-
Others	71 (45.2)	65 (45.5)	0.97	1.01 (0.64–1.59)	16 (66.7)	0.05	2.42 (0.98–5.99)	49 (41.2)	0.50	0.85 (0.52–1.37)
CC/GA	3 (1.9)	0	0.25	-	0	1.00	-	0	0.26	-
CT/GG	4 (2.6)	1 (0.7)	0.37	0.28 (0.03–2.52)	0	1.00	-	1 (0.8)	0.39	0.31 (0.03–2.81)
CT/GA	53 (33.8)	55 (38.5)	0.59	1.14 (0.70–1.86)	15 (62.5)	0.02*	3.04 (1.21–7.66)	40 (33.6)	0.78	0.93 (0.55–1.56)
TT/GG	1 (0.6)	0	1.00	-	0	1.00	-	0	1.00	-
TT/GA	1 (0.6)	1 (0.7)	1.00	1.10 (0.07–17.93)	0	1.00	-	1 (0.8)	1.00	1.23 (0.08–20.00)
TT/AA	9 (5.7)	8 (5.6)	0.97	0.98 (0.36–2.67)	1 (4.2)	1.00	1.19(0.13–10.67)	7 (5.9)	0.93	0.96 (0.34–2.70)
rs6269 A > G; rs4818 C > G										
AA/CC	52 (33.1)	61 (42.7)	-	-	10 (41.7)	-	-	51 (42.9)	-	-
Others	105 (66.9)	82 (57.3)	0.09	0.67 (0.42–1.06)	14 (58.3)	0.41	0.69 (0.29–1.67)	68 (57.1)	0.10	0.66 (0.40–1.08)
AA/CG	4 (2.6)	1 (0.7)	0.19	0.21 (0.02–1.97)	0	1.00	-	1 (0.8)	0.37	0.26 (0.03–2.36)
AG/CG	76 (48.4)	64 (44.8)	0.19	0.72 (0.44–1.18)	12 (50.0)	0.67	0.82 (0.33–2.04)	52 (43.7)	0.18	0.70 (0.41–1.18)
AG/CC	1 (0.6)	3 (2.1)	0.63	2.56 (0.26–25.34)	0	1.00	-	3 (2.5)	0.62	3.06 (0.31–30.38)
GG/CC	2 (1.3)	0	0.22	-	0	1.00	-	0	0.50	-
GG/CG	2 (1.3)	1 (0.7)	0.60	0.43 (0.04–4.84)	0	1.00	-	1 (0.8)	1.00	0.51 (0.05–5.80)
GG/GG	20 (12.7)	13 (9.1)	0.14	0.55 (0.25–1.22)	2 (8.3)	0.72	0.52 (0.11–2.58)	11 (9.2)	0.17	0.56 (0.24–1.29)
6269 A > G; rs4633 C > T; rs4818 C > G; rs4680 G > A;										
1. L/L	20 (12.7)	29 (20.3)	0.10	0.47 (0.19–1.17)	2 (8.3)	1.00	1.05 (0.13–8.24)	27 (22.7)	0.11	0.47 (0.19–1.18)
2. L/M	20 (12.7)	23 (16.1)	0.27	0.60 (0.24–1.50)	7 (29.2)	0.26	0.30 (0.06–1.63)	16 (13.4)	0.64	0.79 (0.30–2.10)
3. M/M	9 (6.3)	8 (5.6)	0.67	0.77 (0.24–2.52)	1 (4.2)	1.00	0.95 (0.08–11.87)	7 (5.9)	0.74	0.81 (0.24–2.76)
4. H/L	45 (31.5)	33 (23.1)	0.87	0.93 (0.40–2.15)	4 (16.7)	1.00	1.18 (0.20–7.02)	29 (24.4)	0.96	0.98 (0.42–2.32)
5. H/M	29 (20.3)	30 (21.0)	0.35	0.66 (0.28–1.58)	8 (33.3)	0.30	0.38 (0.07–2.00)	22 (18.5)	0.70	0.83 (0.34–2.07)
6. H/H	19 (14.0)	13 (9.1)	-	-	2 (8.3)	-	-	12 (10.1)	-	-
7. Rare	15 (10.5)	7 (4.9)	0.51	0.68 (0.22–2.14)	0	0.50	-	6 (5.0)	0.45	0.63 (0.19–2.08)
8. H carrier (4 + 5 + 6)	93 (65.0)	76 (53.2)	-	-	14 (58.3)	-	-	63 (52.9)	-	-
9. L carrier (1 + 2 + 4)	85 (59.4)	85 (59.4)	0.35	1.22 (0.80–1.88)	13 (54.2)	0.97	1.02 (0.45–2.28)	72 (60.5)	0.33	1.25 (0.80–1.96)
10. other (1 + 2 + 3)	49 (31.2)	60 (41.6)	0.10	1.50 (0.92–2.43)	10 (41.7)	0.50	1.36 (0.56–3.28)	50 (42.0)	0.11	1.51 (0.91–2.50)

*CI* confidence interval, *EOPD* early onset PD, *LOPD* late onset PD, *OR* odds ratio

*L* low activity haplotype -A_C_C_G, *M* medium activity haplotype -A_T_C_A, *H* high activity haplotype -G_C_G_G; *P* values calculated in relation to each SNP normal genotype and allele frequency (rs6269 AA, allele A; rs4633 CC, allele C; rs4818 CC, allele C; rs4680 GG, allele G; rs4633-rs4680 CC/GG; rs6269-s4818 AA/CC) as a referent genotype; four SNPs: H/H (6) as a referent genotype [(1–5, 7), H carriers (8) as a referent genotype (9, 10); and L carriers (9) as a referent genotype (11). When more than 20% of the cell numbers were missing, or when the expected number of cases was less than 1.0 in a cell, Fisher’s exact test was performed. *P  0.05

In the subset analysis (Table 2), the frequencies of rs4633 T carriers (*P* = 0.03, odds ratio (OR) = 2.68, 95% confidence interval (CI): 1.06–6.47), rs4680 A carriers (*P* = 0.02, OR = 2.76, 95% CI: 1.12–6.82) and linked rs4633-rs4680 T/A carriers (*P* = 0.05, OR = 2.42, 95% CI: 0.98–5.99) were significantly higher in the EOPD group than in the control group. No significant difference in the SNPs or haplotypes was observed between the LOPD group and controls. No significant difference in the four combined SNPs was observed between the EOPD or LOPD groups and the control groups.

### COMT SNPs influenced PD disease severity, levodopa treatment response and wearing-off phenomenon

The clinical characteristics of the patients with PD who were carriers of different SNPs are listed in Table 3. After adjusted for sex and gender, for rs4633, patients with the TT genotype had a higher H&Y stage (*P* = 0.007 among groups; *P* = 0.006 vs CC, *P* = 0.009 vs CT, respectively), younger age onset (*P* = 0.01 among groups; *P* = 0.03 vs CC), longer disease duration (*P* = 0.005 among groups; *P* = 0.005 vs CC), higher UPDRS scores (*P* = 0.006 among groups; *P* = 0.03 vs CC, *P* = 0.005 vs CT, respectively) (particularly on Part II (*P* = 0.002 among groups; *P* = 0.004 vs CC, *P* = 0.001 vs CT, respectively) and Part III, (*P* = 0.01 among groups; *P* = 0.02 vs CT), higher LED (*P* = 0.005 among groups; *P* = 0.009 vs CC) and more “wearing-off” symptoms (*P* = 0.008 among groups; *P* = 0.006 vs CC) than patients with the other genotypes, indicating that patients with the TT genotype of rs4633 presented more advanced disease stages, required higher dosages of drugs and had more fluctuations than patients with the other two genotypes. For rs4680, patients with the AA genotype also had a higher H&Y stage (*P* = 0.01 among groups; *P* = 0.01 vs GG, *P* = 0.02 vs GA, respectively), younger age onset (*P* = 0.01 among groups; *P* = 0.02 vs GG), longer disease duration (*P* = 0.005 among groups; *P* = 0.006 vs GG), higher LED (*P* = 0.007 among groups; *P* = 0.012 vs GG), higher UPDRS scores (*P* = 0.04 among groups; *P* = 0.03 vs GA) (particularly on Part II (*P* = 0.02 among groups; *P* = 0.02 vs GG, *P* = 0.02 vs GA, respectively)) and more “wearing-off” symptoms (*P* = 0.009 among groups; *P* = 0.008 vs GG) than patients with the other genotypes. These also suggested that the patients with the AA genotype of rs4680 may more easily develop a more severe form of the disease, require larger quantities of drugs and had more fluctuations. For rs4633-4680 (Table 4), patients with the TT/AA genotypes had a higher H&Y stage (*P* = 0.03 among groups; *P* = 0.02 vs CC/GG, *P* = 0.03 vs CT/GA, respectively), younger age onset (*P* = 0.03 among groups; *P* = 0.045 vs CC/GG), longer disease duration (*P* = 0.02 among groups; *P* = 0.01 vs CC/GG), higher UPDRS scores (*P* = 0.047 among groups; *P* = 0.046 vs CT/GA) (particularly on Part II, *P* = 0.03 among groups; *P* = 0.049 vs CC/GG, *P* = 0.02 vs CT/GA, respectively), more “wearing-off” symptoms (*P* = 0.02 among groups; *P* = 0.04 vs CC/GG) and higher LED (*P* = 0.01 among groups; *P* = 0.01 vs CC/GG) than patients without complete variants, indicating that patients who were simultaneous carriers of the TT genotype of rs4633 and AA genotype of rs4680 also showed the same tendency to develop a more severe illness and to experience poor drug treatment effects.

**Table 3:** Relationships between clinical features of patients and single SNPs

Characteristics	Total	rs4633 C > T				rs4680 G > A				rs6269 A > G				rs4818 C > G			
		CC	CT	TT	*P*	GG	GA	AA	*P*	AA	AG	GG	*P*	CC	CG	GG	*P*
n	143	78	56	9		79	56	8		62	67	14		64	66	13	
H&Y stage^b^	2.0 ± 1.0	2.0 ± 1.5	2.0 ± 1.0	3.0 ± 0.5	0.007**	2.0 ± 1.5	2.0 ± 1.0	2.8 ± 0.5	0.01*	2.5 ± 1.0	2.0 ± 1.0	2.0 ± 0.8	0.07	2.5 ± 1.0	2.0 ± 1.0	2.0 ± 0.8	0.09
Age onset (years)^a^	59.2 ± 9.2	60.7 ± 9.2	57.5 ± 9.4	57.3 ± 5.6	0.01*	60.7 ± 9.2	57.6 ± 9.4	56.8 ± 5.7	0.01*	59. 5 ± 9.5	58.828.6	60.3 ± 10.6	0.80	59.6 ± 9. 5	58.6 ± 8.6	60.8 ± 10.9	0.89
Disease duration (years)^b^	6.0 ± 5.0	6.0 ± 4.0	6.0 ± 5.8	9.0 ± 7.5	0.005**	6.0 ± 4.0	6.0 ± 6.0	8.5 ± 9.5	0.005**	6.0 ± 5.3	6.0 ± 5.0	7.5 ± 7.3	0.73	6.0 ± 5.0	6.0 ± 5.0	8.0 ± 8.5	0.75
LED (mg)^a^	448.8 ± 327.4	386.2 ± 287.5	496.8 ± 358.0	693.2 ± 322.6	0.005**	389.5 ± 287.2	497.7 ± 358.5	692.4 ± 344.8	0.007**	475.3 ± 399.2	425.0 ± 241.7	445.5 ± 346.5	0.56	469.7 ± 396.5	435.0 ± 243.6	416.4 ± 342.2	0.59
Wearing-off (yes, %)	43 (30.1)	18 (23.1)	19 (33.9)	6 (66. 7)	0.008**	18 (22. 8)	20 (35.7)	5 (65.1)	0.009**	20 (32.3)	17 (25.4)	6 (42.9)	0.94	20 (31.3)	18 (27.3)	5 (38.5)	0.87
Dyskinesia (yes, %)^b^	18 (12.6)	7 (9.0)	9 (16.1)	2 (22.2)	0.12	7 (8.9)	9 (16.1)	2 (25.0)	0.09	8 (12.9)	7 (10. 5)	3 (21.4)	0.76	8 (12.5)	7 (10.6)	3 (23.1)	0.67
UPDRS Part I score ^b^	2.0 ± 3.0	2.0 ± 3.0	3.0 ± 3.0	2.0 ± 3.0	0.92	2.0 ± 3.0	3.0 ± 3.0	2.0 ± 3.5	0.91	2.0 ± 3.0	2.0 ± 3.0	3.0 ± 3.0	0.41	2.0 ± 4.0	2.0 ± 3.0	3.0 ± 3.0	0.31
UPDRS Part II score ^b^	8.0 ± 7.0	8.5 ± 9.0	8.0 ± 7.3	13.0 ± 10.0	0.002**	8.0 ± 9.0	8.0 ± 6.8	13.0 ± 7.8	0.02*	8.0 ± 7.0	8.0 ± 8.0	11.0 ± 7.3	0.48	8.0 ± 7.0	9.0 ± 7.0	9.0 ± 7.5	0.70
UPDRS Part III score ^a^	24.7 ± 12.1	25.9 ± 11.9	21.8 ± 11.6	32. 8 ± 12.3	0.01*	25.8 ± 11.9	22.4 ± 12.1	31.0011.9	0.06	25.3 ± 12.3	24.2 ± 12.1	24.6 ± 11.6	0.82	24.8 ± 12.3	24.4 ± 12.3	26.2 ± 10.3	0.96
UPDRS Part IV score ^b^	3.0 ± 2.0	3.0 ± 2.0	2.0 ± 3.0	5.0 ± 3.0	0.10	3.0 ± 2.0	2.0 ± 3.8	4.5 ± 4.0	0.13	3.0 ± 4.0	2.0 ± 3.0	3.0 ± 2.0	0.98	3.0 ± 4.0	2.5 ± 3.0	3.0 ± 2.0	0.96
UPDRS total score^a^	40.0 ± 17.7	41.0 ± 17.4	36.3 ± 16.5	54.8 ± 20.2	0.006**	40.8 ± 17.3	37.1 ± 17.3	52.0 ± 19.7	0.04*	40.7 ± 18.4	38.8 ± 17.4	42.6 ± 15.8	0.79	40.2 ± 18.8	39.0 ± 17.1	44.1 ± 15.5	0.81
NMS score^b^	6.0 ± 6.0	6.0 ± 6.0	6.0 ± 4.8	7.0 ± 9.5	0.60	6.0 ± 6.0	6.5 ± 4.8	6.5 ± 10.8	0.68	7.0 ± 6.3	6.0 ± 6.0	6.5 ± 5.5	0.57	7.0 ± 7.8	6.0 ± 5.3	6.0 ± 6.0	0.38
HAMA score^b^	4.0 ± 6.0	4.0 ± 6.0	5.0 ± 4.5	4.0 ± 14.0	0.48	4.0 ± 6.0	5.0 ± 4.8	4.5 ± 13.3	0.40	5.0 ± 6.0	4.0 ± 4.0	6.0 ± 7.0	0.85	5.0 ± 6.0	4.0 ± 4.0	6.0 ± 6.0	0.93
HAMD score^b^	3.0 ± 4.0	2.0 ± 5.0	4.0 ± 4.0	4.0 ± 6.0	0.40	2.0 ± 5.0	4.0 ± 4.0	3.0 ± 6.5	0.20	2.0 ± 6.3	3.0 ± 4.0	2.0 ± 3.8	0.14	2.0 ± 7.0	3.0 ± 4.0	2.0 ± 2.0	0.41
MMSE score^b^	28.0 ± 3.0	28.0 ± 3.0	28.0 ± 3.0	27.0 ± 4.0	0.49	28.0 ± 3.0	28.0 ± 3.0	27.5 ± 3.0	0.54	28.0 ± 3.0	28.0 ± 3.0	28.0 ± 3.5	0.12	28.0 ± 3.0	28.0 ± 3.0	28.0 ± 3.5	0.13

Data were analyzed with analysis of variance of factorial design with age and sex as covariates

Binary logistic regression model was used to analyze the relation between SNP and motor complication (wearing-off phenomenon and dyskinesia), with age and sex as covariates

*P < 0.05 **P < 0.01

^a^Values are expressed as the mean ± SD

^b^Values are expressed as the median ± IQ

**Table 4:** Demographic and clinical features of patients with two combined SNPs

Characteristics	rs4633 C > T; rs4680 G > A					rs6269 A > G; rs4818 C > G				
	CC/GG	CT/GA	TT/AA	Rare	*P*	AA/CC	AG/CG	GG/GG	Rare	*P*
n	78	55	8	2		61	64	13	5	
H&Y stage^b^	2.0 ± 1.5	2.0 ± 1.0	2.8 ± 0.5	2.50 ± 1.0	0.03*	2.5 ± 1.0	2.0 ± 1.0	2.0 ± 0.8	2.0 ± 1.5	0.12
Age onset (years)^a^	60.7 ± 9.2	57.5 ± 9.5	56.8 ± 5.7	58.0 ± 5.7	0.03*	59.4 ± 9.6	58.6 ± 8.8	60.8 ± 10.9	60.8 ± 5.1	0.71
Disease duration (years)^b^	6.0 ± 4.0	6.0 ± 6.0	8.5 ± 9.5	9.0 ± 4.0	0.02*	6.0 ± 5.5	6.0 ± 5.3	8.0 ± 8.5	4.0 ± 3.5	0.67
LED (mg)^a^	386.2 ± 287.5	494.0 ± 360.7	692.4 ± 344.8	675.0 ± 35.4	0.01*	479.0 ± 401.5	431.8 ± 241.4	416.4 ± 342.2	448.8 ± 327.4	0.67
Wearing-off (yes, %)	18 (23.1)	19 (34.6)	5 (62.5)	1(50.0)	0.02*	20 (32.8)	17 (26. 6)	5 (38.5)	1 (20.0)	0.87
Dyskinesia (yes, %)	7 (9.0)	9 (16.4)	2 (25.0)	0	0.07	8 (13.1)	7 (10.9)	3 (23.1)	0	0.52
UPDRS Part I score^b^	2.0 ± 3.0	3.0 ± 3.0	2.0 ± 3.5	3.0 ± 0.0	0.98	2.0 ± 4.0	2.0 ± 3.0	3.0 ± 3.0	3.0 ± 5.5	0.50
UPDRS Part II score^b^	8.5 ± 9.0	8.0 ± 6.0	13.0 ± 7.8	13.5 ± 17.0	0.03*	8.0 ± 7.0	8.5 ± 7.0	9.0 ± 7.5	10.0 ± 13.0	0.77
UPDRS Part III score^a^	25.9 ± 11.9	21.9 ± 11.7	31.0 ± 11.9	32.0 ± 21.2	0.08	25.1 ± 12.3	24.5 ± 12.2	26.2 ± 10.3	19.4 ± 14. 9	0.83
UPDRS Part IV score^b^	3.0 ± 2.0	2.0 ± 3. 0	4.5 ± 4.0	4.0 ± 2.0	0.24	3.0 ± 3.5	2.0 ± 2.8	3.0 ± 2.0	2.0 ± 4.0	0.78
UPDRS total score^a^	41.0 ± 17.4	36.4 ± 16.6	52.0 ± 19.7	52.5 ± 34.7	0.047*	40.5 ± 18.6	39.1 ± 17.2	44.1 ± 15.5	34.6 ± 20.4	0.86
NMS score^b^	6.0 ± 6.0	6.0 ± 4.0	6.5 ± 10.8	6.5 ± 9.0	0.85	7.0 ± 6.5	6.0 ± 5.75	6.0 ± 6.0	9.0 ± 11.0	0.46
HAMA score^b^	4.0 ± 6.0	5.0 ± 5.0	4.5 ± 13.3	3.0 ± 4.0	0.29	5.0 ± 6.0	4.0 ± 4.0	6.0 ± 6.0	8.0 ± 12.5	0.20
HAMD score^b^	2.0 ± 5.0	4.0 ± 4.0	3.0 ± 6.5	2.0 ± 4.0	0.56	2.0 ± 6.5	3.0 ± 4.0	2.0 ± 2.0	7.0 ± 7.5	0.86
MMSE score^b^	28.0 ± 3.0	28.0 ± 3.0	27.5 ± 3.0	26.5 ± 3.0	0.72	28.0 ± 3.0	28.0 ± 3.0	28.0 ± 3.0	29.0 ± 2.0	0.37

Data were analyzed with analysis of variance of factorial design with age and sex as covariates

Binary logistic regression model was used to analyze the relation between SNP and motor complication (wearing-off phenomenon and dyskinesia), with age and sex as covariates

*P < 0.05, ^a^Values are expressed as the mean ± SD; ^b^Values are expressed as the median ± IQ

For rs6269, rs4818 and rs6269-rs4818 (Tables 3 and 4), no significant differences in clinical features, such as age of onset, duration of the disease, H&Y stage, LED, or the UPDRS, NMS, HAMA, HAMD and MMSE scores, were observed. For the four combined functional SNPs (rs6269, rs4633, rs4818, and rs4680) (data not shown), no significant differences in the clinical features, such as age of onset, duration of the disease, H&Y stage, LED, or the UPDRS, NMS, HAMA, HAMD and MMSE scores, were observed.

## Discussion

The COMT enzyme is a natural candidate that has been implicated in the pathogenesis of PD. In this study, we investigated the association between functional COMT haplotypes and PD susceptibility. We found that the frequencies of rs4633 T carriers, rs4680 A carriers and rs4633-rs4680 T/A carriers were significantly higher in the EOPD group than in the healthy control group, suggesting that rs4633 and rs4680 polymorphisms are associated with susceptibility to EOPD. Considering the sample size is small, larger cohort studies focus EOPD patients are needed. However, no other significant difference in the risk of the individual COMT gene variants for PD pathogenesis was observed. The results of previous studies addressing the possible association between SNPs in the COMT gene and the risk of developing PD are controversial, particularly for rs4680, which primarily determines COMT activity. One meta-analysis examined 24 studies of 9,719 patients with PD and 14,634 controls and concluded that the rs4680 polymorphism is not a major determinant of the risk for PD [4]. Based on another meta-analysis, the Val158Met polymorphism may be a risk factor associated with Parkinson’s disease in Asian rather than Caucasian populations [16]. Chuan, Gao [17] examined 13 studies including 1,834 patients and 2,298 controls and showed that the Val158Met polymorphism may be associated with PD in Japanese rather than Chinese populations. In conclusion, there is a remarkable heterogeneity in COMT gene variants in different ethnic groups.

Moreover, one study identified a decreased risk for PD in homozygous carriers of the COMT rs4680G and rs4633C alleles [12], whereas other studies focusing on the rs6269 [1], rs4818 [1, 18] and rs4633 SNPs [1, 19] did not detect a significant influence on PD susceptibility. However, Kimchi-Sarfaty [20] suggested that the determination of haplotypes is more appropriate than SNPs for the analysis of genetic variations in patients with pain, and Nackley, Shabalina [11] found that COMT haplotypes might have more influence on COMT activity than single SNPs. Few studies have focused on the four linked SNPs (rs6269, rs4818, rs4680 and rs4633) regarding pain intensity [21] or cognitive impairment in patients with PD [12]. Studies from Portland [1, 12] (including 322 patients with PD and 357 controls) did not demonstrate an association between functional COMT haplotypes in patients with PD and the controls; the frequencies of low (A_C_C_G) and medium (A_T_C_A) or without high (G_C_G_G) activity haplotypes tended to be slightly lower among patients with PD than among the controls. Unfortunately, our results did not identify an association of the four-combined functional COMT SNPs with patients and controls. The frequencies of low and without high haplotypes tended to be slightly higher among patients with PD, although the difference was not significant. Considering the ethnic factors, this result requires further evaluation.

In our results, both the linkage of rs6269-rs4818 and rs4680-rs4633 in the PD group were higher than that in the control group, such as D’ = 1.00 for rs4680 and rs4633 in control group, D’ = 0.91 for rs4680 and rs4633 in PD group. While according to the online data from the International HapMap Project (http://hapmap.ncbi.nlm.nih.gov/index.html.en) in 2016, the linkages of rs4633 with rs4680 are D’ = 1.0, LOD = 14.07, *r*2 = 0.887, and the linkage of rs6269 with rs4818 has not been shown in Chinese people. In particular, our results were different from data of Oriental individuals concluded from 1000 genome datasets in 2015 [4]. These data of the Oriental individuals were concluded from the northern and southern part in China (Beijing, Yunnan, Hunan and Fujian), while Shanghai, as the eastern part of China, was not involved in that program (http://www.internationalgenome.org/cell-lines-and-dna-coriell). According to the different geographic and host genetic factors, larger sample sizes and multi-center studies are needed in the future. Although no report has yet addressed the issue of dopamine catabolism in relation to the PD risk using a two-SNP model, we speculated that the difference might be associated with PD progression.

The patients with the T allele of rs4633 or the A allele of rs4680 and the TT/AA alleles of the two linked SNPs rs4633-rs4680 had a tendency to experience the “wearing-off” phenomenon, had a longer disease duration and larger daily LED and had higher UPDRS scores, suggesting that patients with rs4633 and rs4680 polymorphisms had more severe disease. Only one study focused on the association of rs4633 with medication and reported that the rs4633 polymorphism was associated with the UPDRS score but not with L-dopa medication [19]. Both rs4633 TT and rs4680 AA encode the low activity COMT enzyme, which may decrease COMT activity and dopamine degradation [8, 22]. The rs4680 SNP has been an area of intense research for several years. Our results were similar to those of other studies. A meta-analysis published in 2015 concluded that allele A of rs4680 was correlated with a risk of PD “wearing-off” [23]. As shown in the study by Watanabe, Harada [22], the “wearing-off” phenomenon tended to occur in patients with PD carrying the COMT rs4680 (AA) SNP. The authors proposed that decreased COMT activity might result in increased neuromelanin metabolism, which might subsequently promote the formation of cytotoxic radicals released upon neuromelanin interactions, contributing to neuronal degeneration. The possible effects of COMT SNPs on PD-correlated neuropharmacological variables are controversial. Bialecka, Kurzawski [1] reported a study of COMT haplotypes in Poland that did not identify a significant impact of COMT haplotypes on the development of the “wearing-off” phenomenon, longer disease duration and larger LED. Hao, Shao [9] found that the rs4680 GG allele may be a risk factor for the “wearing-off” phenomenon. The reasons for the discrepancy with our results may be that the patients involved in our study presented different disease severities. The mean disease duration of our patients was 6.0 years, which was longer than the 3 years of the patients in the study of Hao, Shao [9]. The mean disease duration of the patients in the study by Watanabe, Harada [22] was 9.4 years, which established the same conclusion as our study. In patients with low COMT activity, the prolonged duration and increased quantities of levodopa and dopamine in the plasma may lead to an increased accumulation, which may accelerate the neurodegenerative process. In addition to the “wearing-off” phenomenon, a recent prospective study showed that the homozygosity in the AA allele of the COMT Val158Met polymorphism increases the risk of dyskinesia, which also suggests that the AA genotype may decrease L-dopa metabolism [24]. Our study did not arrive at the same conclusion because of the limited number of patients who presented dyskinesia. Finally, there were no significant effects of rs6269 and rs4818 on the morbidity and pharmacotherapy of PD, and the results are consistent with other currently available studies [4].

However, our results concerning the association of COMT SNPs with PD susceptibility and medication were based on a small sample size, and further investigations, particularly a larger cohort study including patients with different disease severities, should investigate the role of combined functional COMT haplotypes in Chinese Han patients. Additionally, the limitation of technology (e.g., low throughput, only sequenced by segments, needed manual comparison) used in this research could not be ignored, and the novel technologies of high-throughput sequencing become more popular and easily available in the future.

## Conclusions

In conclusion, we provide the first report highlighting the possible association of functional COMT haplotypes with the risk of PD in Chinese patients. The combined COMT genotype also showed a possible influence on the motor response to levodopa and disease severity, particularly the duration, the “wearing-off” phenomenon, daily LED and UPDRS in patients with PD, which may be useful in instituting individualized therapy for patients with PD.

## Additional file

Additional file 1: Table S1. Characteristics of the genotyped COMT SNPs. **Table S2.** Demographic and clinical features of functional COMT haplotype. (DOCX 19 kb)

## Abbreviations

CNS: Central nervous system
COMT: Catechol-O-methyltransferase
EOPD: Early onset PD
H&Y stage: Hoehn and Yahr stage
HAMA: Hamilton Anxiety Scale
HAMD: Hamilton Depression Scale
LD: Linkage disequilibrium
LED: Levodopa equivalent doses
LOPD: Late onset PD
MMSE: Mini Mental State Examination
MSA: Multiple system atrophy
NMS-Quest: Non-Motor Symptoms questionnaire
OR: Odds ratio
PCR: Polymerase chain reaction
PD: Parkinson’s disease
PSP: Progressive supranuclear palsy
SD: Standard deviation
SNP: Single nucleotide polymorphisms
UPDRS: Unified Parkinson’s Disease Rating Scale
