# Supplementary material for: Roles of functional catechol-O-methyltransferase genotypes in Chinese patients with Parkinson’s disease
Source: Transl Neurodegener. 2017 Apr 26;6:11. doi: 10.1186/s40035-017-0081-9 (PMC5405521; doi:10.1186/s40035-017-0081-9)
Supplement: Additional file 1: Table S1. — Characteristics of the genotyped COMT SNPs. Table S2. Demographic and clinical features of functional COMT haplotype. (DOCX 19 kb) [file 40035_2017_81_MOESM1_ESM.docx]

**Title:**

Roles of functional catechol-O-methyltransferase genotypes in Chinese patients with Parkinson’s disease

**Journal name:**

Translational Neurodegeneration

**Author:**

Yiwei Qian, Jiujiang Liu, Shaoqing Xu, Xiaodong Yang, Qin Xiao *

**Corresponding author:**

Qin Xiao

Department of Neurology & Institute of Neurology, Ruijin Hospital affiliated to Shanghai JiaoTong University School of Medicine, Shanghai 200025, China

Address: No. 197, Ruijin Er Road, Shanghai, China. 200025

E-mail: [xiaoqin67@medmail.com.cn](mailto:xiaoqin67@medmail.com.cn)

Table S1. Characteristics of the genotyped COMT SNPs

| **SNP** | **Position** | **Location** | **Allele(minor/major)** | **MAF (%)** |
| --- | --- | --- | --- | --- |
| rs6269 | 18329952 | Exon3 | G/A | 37.4 |
| rs4633 | 18330235 | Exon3 | T/C | 26.2 |
| rs4818 | 18331207 | Exon4 | G/C | 36.7 |
| rs4680 | 18331271 | Exon4 | A/G | 26.7 |

Table S2. Demographic and clinical features of functional COMT haplotype

| **characters** | **rs6269 A>G;rs4633 C>T; rs4818 C>G; rs4680 G>A;** | | | | | | | ***P*** |
| --- | --- | --- | --- | --- | --- | --- | --- | --- |
|  | **L/L** | **L/M** | **M/M** | **H/L** | **H/M** | **H/H** | **Rare** |  |
| N | 29 | 23 | 8 | 33 | 30 | 13 | 7 |  |
| Male (%) | 16(55.2%) | 11(47.8%) | 2(25.0%) | 17(51.5%) | 19(63.3%) | 10(76.9%) | 4(57.1%) | 0.23 |
| Age(years) ^a^ | 65.2±9.7 | 66.1±8.4 | 66.2±5.1 | 65.7±8.6 | 62.4±9.7 | 67.6±8.3 | 64.3±4.7 | 0.51 |
| H&Y stage ^b^ | 2.5±0.8 | 2.5±0.5 | 2.8±0.3 | 2.0±0.8 | 2.0±0.5 | 2.0±0.4 | 2.0±1.0 | 0.14 |
| Age of diagnosis(years)^a^ | 60.8±10.2 | 58.8±10.2 | 56.8±5.7 | 60.6±8.1 | 56.4±9.2 | 60.8±10.9 | 60.0±4.9 | 0.40 |
| Disease duration(years)^b^ | 5.0±4.0 | 6.0±6.0 | 8.5±9.5 | 6.0±4.5 | 6.0±4.8 | 8.0±8.5 | 6.0±4.0 | 0.24 |
| LED (mg)^a^ | 354.1±298.8 | 544.7±493.6 | 692.4±344.8 | 400.6±254.3 | 457.1±225.8 | 416.4±342.2 | 467.1±284.8 | 0.07 |
| Wearing-off (yes, %) | 5(17.2%) | 10(43.5%) | 5(62.5%) | 7(21.2%) | 9(30.0%) | 5(38.5%) | 2(28.6%) | 0.07 |
| Dyskinesia (yes, %) | 2(6.9%) | 4(17.4%) | 2(25.0%) | 2(6.1%) | 5(16.7%) | 3(23.1%) | 0 | 0.40 |
| UPDRS Part I score ^b^ | 2.0±3.5 | 2.0±4.0 | 2.0±3.5 | 1.0±2.0 | 3.0±2.3 | 3.0±3.0 | 3.0±4.0 | 0.15 |
| UPDRS Part II score ^b^ | 8.0±8.5 | 7.0±7.0 | 13.0±7.8 | 8.0±7.0 | 8.5±6.5 | 9.0±7.5 | 10.0±15.0 | 0.09 |
| UPDRS Part III score ^a^ | 27.2±12.3 | 20.7±11.5 | 31.0±11.9 | 25.3±12.2 | 22.8±11.6 | 26.2±10.3 | 23.0±16.2 | 0.21 |
| UPDRS Part IV score ^b^ | 3.0±2.5 | 2.0±4.0 | 4.5±4.0 | 2.0±2.5 | 2.5±3.3 | 3.0±2.0 | 3.0±5.0 | 0.55 |
| UPDRS total score ^a^ | 42.4±18.7 | 34.7±16.6 | 52.0±19.7 | 38. 7±17.2 | 38.2±16.3 | 44.1±15.5 | 39.7±23.5 | 0.17 |
| NMS score ^b^ | 7.0±7.5 | 8.0±5.0 | 6.5±10.8 | 5.0±5.5 | 6.0±5.3 | 6.0±6.0 | 9.0±10.0 | 0.50 |
| HAMA score ^b^ | 4.00±7.00 | 5.00±5.00 | 4.50±13.25 | 3.00±4.00 | 5.00±4.25 | 6.00±6.00 | 5.00±7.00 | 0.22 |
| HAMD score ^b^ | 2.00±7.00 | 3.00±7.00 | 3.00±6.50 | 2.00±3.50 | 4.00±2.2.5 | 2.00±2.00 | 4.00±7.00 | 0.14 |
| MMSE score ^b^ | 27.00±5.00 | 28.00±3.00 | 27.50±3.00 | 28.00±3.00 | 28.00±3.25 | 28.00±3.50 | 29.00±3.00 | 0.84 |

L, ‘low activity haplotype’ -A_C_C_G; M, medium activity haplotype-A_T_C_A; H, high activity haplotype-G_C_G_G;

^a^ one-way ANOVA test; ^b^ Kruskal Wallis Test

Supplemental Table 3 multivariate logistic regression of H&Y stage, PD disease duration, UPDRS scores and LED with demographic and clinical features of patients

| **variables** | **UPDRS score** | | | **H&Y stage** | | **disease duration** | | **LED** | |
| --- | --- | --- | --- | --- | --- | --- | --- | --- | --- |
|  | ***P* value** | | **OR(95%CI)** | ***P* value** | **OR(95%CI)** | ***P* value** | **OR(95%CI)** | ***P* value** | **OR(95%CI)** |
| rs4633 | 0.388 | | 2.051(0.401-10.484) | 0.578 | 0.602(0.100-3.609) | 0.499 | 1.812(0.323-10.158) | 0.237 | 0.742 (0.452-1.217) |
| rs4680 |  | |  |  |  |  |  |  |  |
| rs4633&4680 | | |  |  |  |  |  |  |  |
| gender | | 0.033 | 2.032(1.059-3.901) | 0.365 | 0.713(0.344-1.481) | 0.875 | 0.937(0.479-1.830) | 0.013* | 2.199(1.182-4.094) |
| age | | 0.581 | 0.972(0.879-1.075) | 0.151 | 1.085(0.971-1.213) | <.0001*** | 0.449(0.385-0.524) | 0.453 | 0.986(0.950-1.023) |
| H&Y stage | | <.0001*** | 0.087(0.045-0.167) | |  | 0.137 | 0.592(0.296-1.181) | 0.007** | 0.419(0.223-0.789) |
| Disease duration | | 0.150 | 0.906(0.793-1.036) | 0.143 | 0.892(0.765-1.040) | |  | <.0001*** | 0.808(0.734-0.889) |
| Wearing-off | | 0.811 | 1.134(0.405-3.179) | 0.575 | 1.406(0.427-4.626) | 0.221 | 1.942(0.672-5.614) | 0.002** | 3.782(1.655-8.643) |
| Dyskinesia | | 0.086 | 0.376(0.123-1.15) | 0.585 | 1.443(0.387-5.377) | 0.370 | 1.685(0.538-5.280) | 0.601 | 1.322(0.465-3.762) |
| UPDRS score | | |  | <.0001*** | 0.891(0.862-0.921) | 0.372 | 0.986(0.956-1.017) | 0.005** | 1.042(1.013-1.073) |
| NMS score | | 0.469 | 0.963(0.868-1.067) | 0.558 | 0.966(0.860-1.085) | 0.818 | 0.987(0.887-1.099) | 0.867 | 0.992(0.900-1.093) |
| HAMD score | | 0.037 * | 0.876(0.773-0.992) | 0.124 | 1.123(0.969-1.300) | 0.919 | 0.993(0.872-1.131) | 0.813 | 0.986(0.877-1.108) |
| HAMA score | | 0.355 | 0.952(0.857-1.057) | 0.430 | 0.953(0.845-1.074) | 0.339 | 1.055(0.945-1.179) | 0.762 | 0.986(0.898-1.082) |
| MMSE score | | 0.002 ** | 1.105(1.037-1.176) | 0.571 | 0.979(0.911-1.053) | 0.567 | 1.021(0.951-1.096) | 0.047* | 1.062(1.001-1.126) |
| LED | | 0.643 | 0.999(0.995-1.003) | 0.950 | 1.000(0.996-1.004) | 0.069 | 0.996(0.992-1.000) |  |  |

Rs4680 and rs4633&4680 have been set to 0, since the variables are a linear combination of rs4633.

95% CI: 95% confidence interval,****P***<0.05, *****P***<0.01, ******P***<0.001

Supplemental Table 4.Logistic regression modal of the association between wearing-off phenomenon and related variables

| **variable** | **Univariate analysis** |  | **Multivariate analysis** |  |
| --- | --- | --- | --- | --- |
|  | ***P* value** | **OR(95% CI)** | ***P* value** | **OR(95% CI)** |
| Rs4633 | 0.011* | 0.468(0.261-0.841) | 0.659 | 2.174(0.069-68.494) |
| Rs4680 | 0.013* | 0.471(0.260-0.855) | 0.571 | 0.357(0.010-12.652) |
| Rs4633&rs4680 | 0.011* | 0.933(0.885-0.984) | - | - |
| Gender | 0.152 | 1.720(0.819-3.612) | 0.988 | 1.009(0.311-3.272) |
| Age | 0.003** | 1.071(1.024-1.120) | 0.000*** | 1.185(1.088-1.291) |
| H&Y stage | 0.000*** | 0.282(0.148-0.536) | 0.986 | 0.989(0.276-3.538) |
| Disease duration | 0.000*** | 0.772(0.692-0.862) | 0.012* | 0.800(0.671-0.953) |
| UPDRS I score | 0.003** | 0.762(0.637-0.911) | 0.063 | 0.673(0.444-1.021) |
| UPDRS II score | 0.000*** | 0.866(0.803-0.934) | 0.022* | 0.787(0.642-0.966) |
| UPDRS III score | 0.018* | 0.963(0.933-0.994) | 0.519 | 1.025(0.950-1.106) |
| UPDRS IV score | 0.000*** | 0.652(0.537-0.793) | 0.039* | 0.711(0.515-0.983) |
| UPDRS score | 0.000*** | 0.959(0.937-0.981) | - | - |
| NMS score | 0.031* | 0.913(0.840-0.992) | 0.077 | 1.264(0.975-1.639) |
| HAMD score | 0.056 | 0.926(0.856-1.002) | 0.967 | 1.004(0.820-1.231) |
| HAMA score | 0.122 | 0.948(0.887-1.014) | 0.569 | 1.052(0.883-1.254) |
| MMSE score | 0.231 | 1.039(0.976-1.105) | 0.323 | 0.944(0.841-1.059) |
| LED | 0.000*** | 0.994(0.993-0.997) | 0.010** | 0.996(0.993-0.999) |

95% CI: 95% confidence interval,****P***<0.05, *****P***<0.01, ******P***<0.001
